# Improved assays to measure and characterize the inducible HIV reservoir

**DOI:** 10.1016/j.ebiom.2018.09.036

**Published:** 2018-10-11

**Authors:** Marta Massanella, Christina Yek, Steven M. Lada, Masato Nakazawa, Neda Shefa, Karissa Huang, Douglas D. Richman

**Affiliations:** a University of California San Diego, La Jolla, CA, USA; b Veterans Affairs San Diego Healthcare System, La Jolla, CA, USA

**Keywords:** HIV reservoir, VOA, Inducible HIV RNA, Latency, Eradication

## Abstract

**Background:**

Improved assays are critical to better characterize the HIV reservoir and to reliably evaluate candidate intervention strategies. Here we describe different methods to quantify the HIV reservoir.

**Methods:**

We developed an optimized quantitative viral outgrowth assay (QVOA) to quantify the frequency of cells harboring replication-competent HIV, which is simpler and more sensitive than classical QVOAs. We also developed new inducible RNA assays that concomitantly measure the frequency of cell-associated [ca-] (gag and tat-rev) and cell-free [cf-] HIV RNA after three days of anti-CD3/CD28 stimulation.

**Findings:**

The median frequency of the infected cells measured after induction was 94 IQR[60–132], 16 IQR [9–29] and 2.9 IQR[1.9–6.8] cells/10^6^ CD4^+^ T-cells for ca-RNA gag and tat-rev, and cf-RNA, respectively. There are a large proportion of transcription-competent proviruses (ca-RNA) that seemed unable to form complete virions (cf-RNA), suggesting post-transcriptional blocks or defective proviruses. Importantly, the median frequency of infected CD4^+^ T-cells as estimated by 3-day inducible cf-RNA assay was not statistically different from the frequency measured by the QVOA (median of 3.3 [1.9–6.2] IUPM). The latently infected cells detected by the inducible cf-RNA assay correlated highly with the QVOA ( r= 0.67, *p* < .001), and both assays were equivalent in 60% of the samples tested, suggesting that most cells induced to produce virions are generating replication-competent virus.

**Interpretation:**

These inducible RNA assays provide more sensitivity and a greater dynamic range for the monitoring of reduction of the reservoir by eradication strategies. Such assays may serve as robust and useful tools for clinical investigations of the HIV reservoir.

## Highlights

• A large proportion of HIV DNA is defective and is unable to produce HIV RNA or replication competent virus after stimulation
• Inducible RNA assays are more sensitive and show a greater dynamic range for the monitoring HIV reservoir decay than viral-outgrowth methods
• Cells producing viral particles after activation are similar in frequency to cells harboring replication competent virus.The other bullets should end with a period.

**Research in context**

HIV persists in infected individuals in a stable pool of CD4 T cells. This HIV reservoir is the major barrier to the eradication of HIV and it has been extremely challenging to accurately quantify the size of the replication-competent reservoir (Eriksson et al, Plos Pathogens 2013). Therefore, improved assays are critical both to better characterize the poorly understood HIV reservoir and to reliably measure candidate intervention strategies.

We describe different methods to quantify the HIV reservoir, including an optimized quantitative viral outgrowth assay (QVOA, the gold-standard to quantify the replication competent reservoir) and novel assays that measure the inducible reservoir. Similar to the Tat/rev Induced Limiting Dilution Assay (Procopio et al, Ebiomedicine 2015), our new inducible RNA assays can measure the frequency of cells with inducible HIV RNA upon stimulation. The great advantage of our assay is that allows for the concomitant measurement of different HIV RNA transcripts and viral production after only three days. Importantly, we show that the quantification of viral production shows comparable performance to our modified QVOA. Importantly, the induction assays described can provide increased sensitivity and a greater dynamic range for the monitoring of reduction of the HIV reservoir by intervention and eradication strategies. The performance characteristics of these assays, including relative sensitivity, precision and specimen requirements, are presented.

Alt-text: Unlabelled Box

## 1 Introduction

Combination antiretroviral therapy (ART) suppresses HIV replication to undetectable plasma levels for prolonged periods; however, it fails to eradicate the virus [[1], [2], [3]]. HIV can persist within a small subset of long-lived, proliferating CD4^+^ T cells infected with integrated latent virus. Targeting and eliminating this pool of latently infected cells is an essential component of the quest for a cure [4]. Strategies aimed at reactivating latent virus, and thereby accelerating the clearance of the latent reservoir, are being investigated [[5], [6], [7], [8], [9]]. Approximately one in 10^5^ to 10^8^ CD4^+^ T cells is latently infected in most patients, with the rate of infection depending on individual variability and when ART was started after infection [10]. These low frequencies highlight the many technical challenges for measuring the HIV reservoir. An assay to measure the HIV reservoir requires sufficient sensitivity and specificity to detect small numbers of HIV-infected cells, and the ability to distinguish between replication competent and incompetent proviruses. Because many assays are close to the limit of detection, the dynamic range that permits the measurement of a reduction of the HIV reservoir by a candidate intervention for eradication is usually quite small. Moreover, the coefficient of variation of assays almost always increases as they approach the limit of detection, making the interpretation more challenging.

The most easy, sensitive and reproducible assays to measure the size of the HIV reservoir are PCR based, which quantify HIV DNA from enriched CD4^+^ T cells [11]. Despite their sensitivity and precision, these assays fail to distinguish between replication competent and defective proviral genomes [[12], [13], [14]]. As such, these assays may overestimate the size of the latent reservoir by 300-fold [11]. The quantitative viral outgrowth assay (QVOA) has been recognized as the “gold-standard” assay for determining the frequency of CD4^+^ T cells harboring replication competent proviruses. This assay applies limiting dilutions of resting CD4^+^ T cells that are activated. This activation reverses latency and reinitiates the production of infectious HIV-1 from the subset of CD4^+^ T cells harboring replication competent proviruses. The viruses that are produced in vitro are propagated in feeder cells, which could be PHA-stimulated CD4^+^ lymphoblasts from HIV-uninfected donors [2,11,[15], [16], [17], [18]] or cell lines such as MOLT-4/CCR5 [19] or SupT1-CCR5 [20]. After two or three weeks, viral outgrowth is assessed by an ELISA assay for HIV-1 p24 antigen or for HIV-1 RNA in the culture supernatant. The frequency of latent infection, expressed as infectious units per million (IUPM) CD4^+^ T cells, is determined using Poisson statistics. Typically, individuals on long term ART exhibit IUPM values between 0.1 and 1 [2,11,15], highlighting that this assay can only detect one infectious unit per hundreds of HIV proviral genomes. It is unclear how much of this low proportion is attributable to replication incompetent proviruses and how much is attributable to suboptimal sensitivity of the current assays due to intact but non-inducible proviruses following activation [12].

Recently, assays of HIV RNA transcripts induced from CD4^+^ lymphocytes have been developed to measure the inducible HIV reservoir [[21], [22], [23]]. These assays have the advantage of reducing the contribution of most defective genomes and not relying on an amplification step with virus propagation by co-culture. These PCR procedures for detecting integrated HIV overestimate the size of the replication competent latent reservoir, however, while viral outgrowth assays underestimate it [12]. The correlation between both assays has not been strong [11,12]. To address this, we increased the sensitivity of the QVOA by optimizing the induction of cell activation [24], improving the culture conditions, and detecting HIV RNA rather than p24 protein. We also developed new assays that measure the frequency of cells expressing inducible cell-associated (ca-RNA) or cell-free (cf-RNA) HIV RNA. Finally, we compared performance of the different assays in samples from 35 HIV-infected, ART-suppressed individuals. Significantly, these assays have potential for application in larger clinical trials and cohort studies as they are robust, sensitive, fast and increase the dynamic range of reservoir size over QVOA assays.

## 2 Methods

### 2.1 Participant samples

Thirty-five HIV-infected individuals on suppressive ART (plasma HIV RNA <50 copies/ml) for at least 1 year and who started ART >1 year after infection were included in the study. All determinations were performed with fully informed written consent from all participants. Subject characteristic is summarized on Table 1.

**Table 1:** Characteristics of the cohort.

	All cohort	Comparison all assays	*p*-Value
	n = 35	n = 19	
Age, years	52 [47–59]	55 [45–61]	0.62
Male, n (%)	31 (88)	17 (89%)	1
Time since EDI, years	18 [11–24]	18 [9–27]	0.88
Time on suppressed, years	4 [1.8–7.8]	6 [1.5–9.8]	0.7
Absolute CD4 T-cell count, cells/μl	624 [479–758]	525 [468–734]	0.41
*EDI: estimated date of infection*			

Peripheral blood mononuclear cells (PBMCs) were isolated using density gradient centrifugation from whole blood (*n* = 29) or leukapheresis (*n* = 6). Total CD4^+^ T cells were enriched from PBMC by negative selection using EasySep CD4 Enrichment kit (Stem Cell Technologies). Enriched CD4^+^ T cells were freshly used for mQVOA (*n* = 32) and inducible RNA assays in limiting dilution (*n* = 35 and *n* = 19 for cf-RNA and ca-RNA, respectively) or bulk (*n* = 15, Supplementary fig. 1). When enough additional enriched CD4^+^ T cells were still available (*n* = 3), cells were cryopreserved to assess assay reproducibility after freezing.

### 2.2 HIV DNA and RNA quantification

DNA and RNA were co-extracted from 5 million freshly isolated PBMCs using the All prep extraction kit (Qiagen). Total HIV DNA (gag) and ca-RNA (gag and tat-rev) were quantified by droplet digital PCR (ddPCR) from extracted DNA or RNA, respectively, as described previously [25]. Copy numbers were normalized to 1 million CD4 T cells as determined by RPP30 (total cell count) and flow cytometry (percentage of CD4 T cells).

### 2.3 Modified quantitative viral outgrowth assay (mQVOA)

Enriched CD4^+^ T cells were serially diluted in a 24-well plate coated with anti-CD3 (Clone SK7) and anti-CD28 (Clone CD28.2) monoclonal antibodies (both from BD Biosciences). Seven serial 3-fold dilutions were performed, usually at a starting concentration of 1 × 10^6^ cells/well (ranging from 1.5 to 0.5 million, depending on cell availability); 6 replicates were performed for each dilution. After two days of stimulation, 200,000 MOLT-4/CCR5 cells were added to each cell culture well (day 0). Cell cultures were split twice weekly; half of cell culture supernatants (500 μl) were collected for analysis at days 7 and 14. These supernatants were spun at 300 *g* for 5 min and frozen at −80 °C until use. HIV-specific magnetic beads were used to extract cf-RNA from supernatants (Aptima HIV-1 kit, Hologic Incorporated). Briefly, supernatants were incubated with 400 μl of Target Capture Reagent (TCR, containing HIV-specific magnetic beads) for 7 min at 80 °C, 30 min at 60 °C and 22 min at 25 °C, After incubation, the magnetic particles were concentrated using a KingFisher instrument (Life Technologies). Extracted HIV RNA was then detected using One-step RT-PCR (Applied Biosystems) for detection of *pol (*limit of detection 50 copies/ml, limit of quantification 6.25 copies/ml) [26]. The number of wells positive for HIV RNA was determined, and the maximum likelihood method was applied to determine IUPM (http://silicianolab.johnshopkins.edu/) [27]. A schema of the mQVOA is depicted in Supplementary fig. 1A.

### 2.4 Inducible RNA assays

Similar to mQVOA, enriched CD4^+^ T cells were serially diluted in a 96-well plate coated with the anti-CD3/CD28 monoclonal antibodies. Eight serial 3-fold dilutions were performed usually at a starting concentration of 0.5 × 10^6^ cells/well (ranging from 1 to 0.25 million cells); between 6 and 12 replicates were performed for each cell-dilution. After 3 days of stimulation in the presence of raltegravir, the cell cultures were collected and spun at 300 *g* for 5 min. The supernatants were collected and frozen directly at −80 °C, while cell pellets were resuspended in Lysis/Binding solution (Magmax RNA extraction kit, Life Technologies) and also stored at −80 °C until use. When enough cells were available, 5 × 10^6^ CD4^+^ T cells were stimulated in bulk for 3 days in a 6-well plate coated with anti-CD3/CD28 antibodies in the presence of raltegravir.

Cf-RNA from supernatants (in dilution [200 μl] or in bulk [600 μl]) was extracted using the Aptima HIV-1 target capture system as described for mQVOA. Ca-RNA was extracted using Magmax RNA extraction kits (Life Technologies) following manufacturer's instructions. Droplet digital PCR (ddPCR, Biorad) was performed to quantify *gag* [28] for cf-RNA, and *gag* (unspliced RNA [usRNA]) [28] and *tat-rev* (multiply spliced RNA [msRNA]) [29] in multiplex for ca-RNA, as previously described [25]. As with mQVOA, positive wells at each dilution of the inducible cf-RNA and ca-RNA assays were counted and the maximum likelihood method was used to calculate the frequency of cells with inducible HIV RNA [30]. A schema of the inducible RNA assay is depicted in Supplementary fig. 1B—C.

### 2.5 Statistical analysis

mQVOA, and inducible cf-RNA and ca-RNA were compared using the Wilcoxon matched-pairs signed rank test or one-way ANOVA. For correlations between values obtained with different assays, log transformed virologic data were normally distributed and compared using the Pearson test. Equivalence was assessed by plotting IUPM mQVOA levels at day 14 against the frequency of HIV infected cells (measured by inducible cf-RNA assay) and computing 95% confidence intervals (CI95) for both variables for each sample. The variables were deemed equivalent when either of the CI95 included the equivalent line with an intercept of 0 and slope of 1. Data analysis was performed using Prism 7.0 and R software (http://www.R-project.org/) [31]. Displays of correlation coefficients were created using Corrplot package software (http://cran.r-project.org/web/packages/corrplot/index.html) [32].

## 3 Results

### 3.1 Optimization of conditions for the modified viral outgrowth assay (mQVOA)

**Fig. 1:**
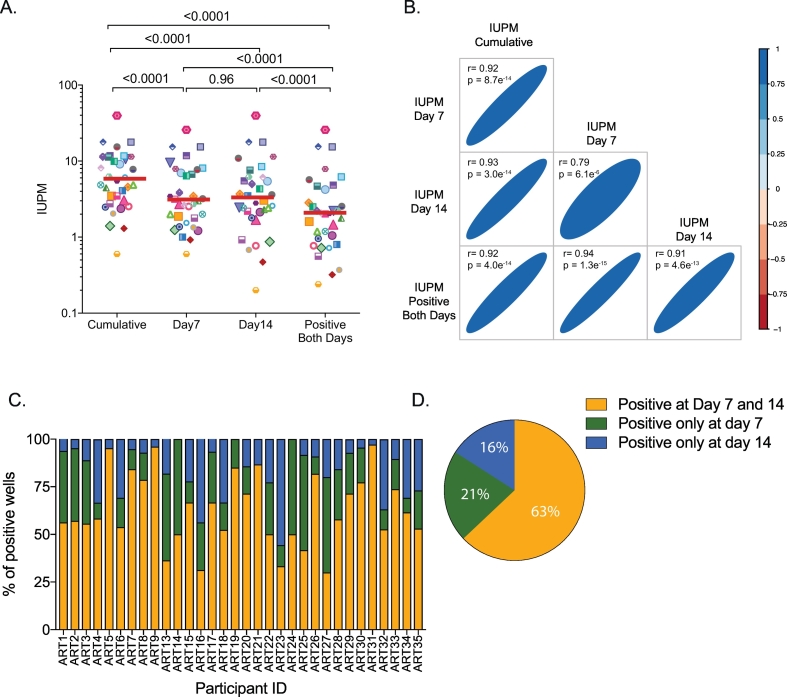
Modified Quantitative viral outgrowth assay (mQVOA). Total CD4^+^ T cells from ART-suppressed individuals (n = 32) were stimulated for 2 days with plate bound anti-CD3/CD28 antibodies, after which feeder cells (MOLT-4/CCR5 cells) were added. Cultures were split twice weekly and culture supernatants were collected at days 7 and 14. (A) Infectious units per million (IUPM) of assayed CD4^+^ T cells were calculated using the online calculator (http://silicianolab.johnshopkins.edu/) at day 7 or 14 and compared to the IUPM for wells positive at both days 7 and 14 (Positive both days IUPM), or for wells positive at either time point (Cumulative IUPM). Log_10_ transformed individual values and median IUPM are shown. One-way ANOVA *p*-values are indicated. (B) Correlation matrix between the different log_10_ transformed IUPM calculated from panel A are plotted. Coefficients of correlation and p-values were obtained from Pearson test. The colour and width of the ellipse show the strength of the correlation between two variables (a narrow ellipse indicates stronger correlation) and tilt the direction. (C) For each individual, we show the percentage of wells considered positive at day 7 and 14 (in yellow), only positive at day 7 (green) or only positive at day 14 (blue). (D) Mean data from panel C are summarized.

We measured the frequency of cells harboring replication competent virus in 32 ART-suppressed subjects using our mQVOA, which assesses the amplification of replication competent virus by measuring cf-RNA in the culture supernatants at days 7 and 14 by RT-PCR (Supplementary Fig. 1A). Since this assay readout has been described to detect the viral production earlier than the quantification by p24 ELISA [19], we calculated the frequencies of replication competent virus using different criteria to determine if we could diminish the culture time. The IUPM at days 7 and 14 were each determined directly from the number of cf-RNA-positive wells. We also estimated the IUPM using the number of cf-RNA positive wells at both time points (days 7 and 14) and compared it to the cumulative IUPM, which was estimated considering all cf-RNA positive wells independent of the time point. We found that there was no statistical difference between the IUPM at day 7 and 14 (median 3.1 [1.6–7.6] and 3.3 [1.9–6.2] IUPM of CD4^+^ T cells, respectively); however, considering only wells that were cf-RNA positive at both time points, the median frequency of cells harboring replication virus was 2.1 [0.8–4.6] IUPM, which was statistically lower than IUPMs at either days 7 or 14 (*p* < .0001 in both cases, Fig. 1A). In contrast, cumulative IUPM showed consistently higher frequencies of replication competent virus compared to all other measures of IUPM (5.8 [2.8–11.4] IUPM CD4^+^ T cells, p < .0001 in all cases, Fig. 1A). The higher values of cumulative IUPM as compared to day 7 and day 14 IUPM likely reflects virus that may not have been sufficiently fit to sustain growth in vitro or virus that may have delayed expansion, respectively. Importantly, all IUPMs highly correlated with each other (Fig. 1B). Among positive wells, the mean proportion of wells that were cf-RNA positive at both time points (days 7 and 14) was 63%, suggesting that a vast majority of the positive wells showed continuous amplification of replication virus (Fig. 1 C and D). A mean proportion of 21% and 16% of all positive wells were cf-RNA positive only at day 7 or day 14, respectively. In the remaining comparative analyses we chose to use day 14 IUPM to represent mQVOA values, as we wanted to include both viruses that continuously expanded in vitro (wells positive at day 7 and 14) and those with slower kinetics that took longer to become detectable (wells positive only at day 14), but not viruses that were unable to sustain growth (wells positive only at day 7).

Since the number of CD4^+^ T cells obtained from a blood draw from a clinical trial can be limited, we wanted to evaluate the minimum number of cells required to maintain the precision and accuracy of the mQVOA assay. We calculated the IUPM using 6, 4, 3, 2 or 1 assay replicates, corresponding to 9.0, 7.5, 6.0, 4.5, 3.0 or 1.5 × 10^6^ CD4^+^ T cells assayed, respectively (Supplementary fig. 2A, left panel). No statistical differences were observed when the IUPM were measured using fewer replicates; however, as expected the CI95 enlarged as the number of replicates diminished and the CI95 was significantly wider when using fewer than 4 replicates compared to the original set up (Supplementary fig. 2B, left panel). Thefore, a minimum of 7.5 × 10^6^ CD4 T cells, distributed in 5 replicates and 3-fold dilutions, should be assayed to provide the best balance of required cell numbers with optimal assay precision and accuracy.

Finally, we evaluated the impact of freezing on these different assays by comparing cryopreserved cells with fresh cells from the same specimen (Supplementary fig. 2C, left panel). We found no statistical difference in the frequency of cells harboring replication competent virus when the mQVOA assay was run with fresh or frozen CD4^+^ T cells.

### 3.2 Concomitant measurement of the frequency of CD4^+^ T cells with inducible us- and ms-RNA, and viral particle production using novel inducible RNA assays

**Fig. 2:**
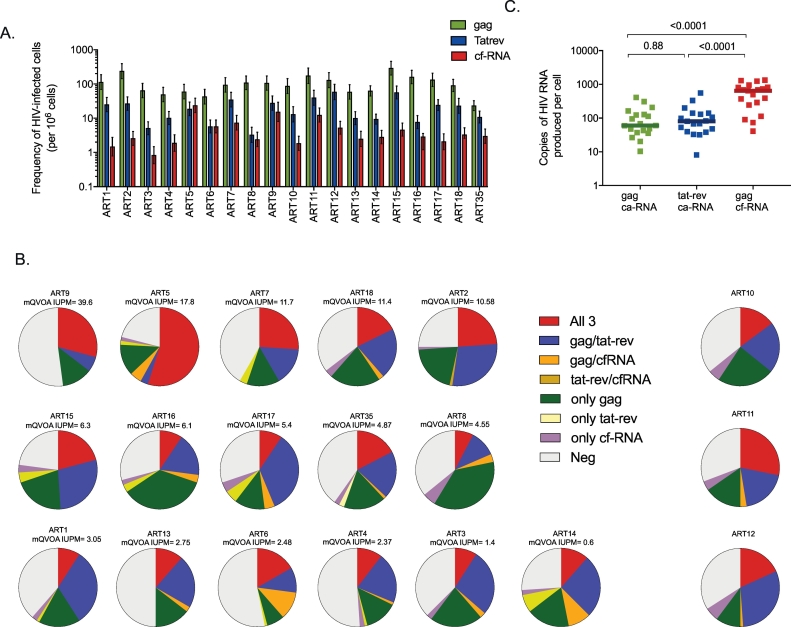
Characterization of Inducible RNA Assays. Limiting dilution of CD4^+^ T cells from ART-suppressed subjects (*n* = 19) were stimulated with anti-CD3/CD28 antibodies for 3 days in the presence of raltegravir. Activated cells as well as culture supernatants were extracted and analyzed for HIV RNA production by ddPCR. The frequencies of the inducible reservoir for each transcript were calculated using the same online calculator as for mQVOA (http://silicianolab.johnshopkins.edu/). (A) Inducible RNA assays can concomitantly quantify in each well the frequency of cells producing ca-RNA, including usRNA gag (green) and msRNA tat-rev (blue) and viral production (cf-RNA, red), as shown in supplemantary figure 3. For each individual, the frequency of cells expressing a given HIV RNA transcript and upper and lower CI95 are plotted. (B) For each individual, the proportion of wells with 3, 2, 1 or 0 RNA transcripts detected in the same well are shown. Pie charts were ordered by IUPM from mQVOA (when available) highlighting the correlation between cf-RNA and mQVOA. (C) Estimation of the copies of HIV RNA usRNA gag, msRNA and viral particles produced per cell in the inducible RNA assay. Total RNA copies from the limiting dilution assay (sum of all positive wells) were normalized by the frequency of cells expressing a given transcript. Log_10_ transformed individual values and medians are shown. One-way ANOVA *p*-values are indicated.

Ex vivo quantification of HIV transcripts (in bulk) revealed that 40% and 90% of the samples were detectable for ca-RNA tat-rev and gag, respectively, and when detectable the values were low (<1 copy and < 30 copies RNA tat-rev and gag /10^6^ CD4 T cells, respectively). Nineteen individuals randomly selected from the cohort were used to run the all of the inducible RNA assays. For that. CD4^+^ T cells were stimulated with anti-CD3/CD28 antibodies in a limiting dilution format in the presence of raltegravir, to avoid new rounds of infection. After 3 days of culture, the RNA from the cells and supernatant were both extracted and quantified to determine the frequency of cells expressing ca-RNA (both unspliced *gag* ca-RNA and multiply spliced *tat-rev* ca-RNA) and cf-RNA (*gag*), respectively (Supplementary Fig. 1B). *gag* ca-RNA was the most common RNA species found in all individuals, followed by *tat-rev* ca-RNA, and finally *gag* cf-RNA (median frequency of 94 IQR [60–132], 16 IQR [[9], [10], [11], [12], [13], [14], [15], [16], [17], [18], [19], [20], [21], [22], [23], [24], [25], [26], [27], [28], [29]] and 3.3 IQR [1.9-6.2] cells expressing HIV RNA per 10^6^ of CD4^+^ T cells, respectively, Fig. 2A). The frequency of cells producing *gag* ca-RNA was 6 fold higher than the frequency of cells producing *tat-rev* ca-RNA. The frequencies of cells producing ca-RNA as compared to those producing viral particles (cf-RNA) were 31 and 5 times higher for *gag* and *tat-rev* ca-RNA respectively.

The inducible RNA assays measure three HIV transcripts (*gag* and *tat-rev* ca-RNA) and virion production (cf-RNA) concomitantly in the same culture well; two examples are depicted in Supplementary fig. 3 and Fig. 2A. A median of 94% [91–96] of all positive wells from all individuals tested were positive for the *gag* ca-RNA transcript, while only 66% [58–70] and 33% [27–51] of all positive wells were positive for the *tat-rev* ca-RNA transcript and cf-RNA, respectively (Fig. 2B). Only a median of 25% [[15], [16], [17], [18], [19], [20], [21], [22], [23], [24], [25], [26], [27], [28], [29], [30], [31], [32], [33], [34], [35], [36]] of all positive wells were positive for all three RNA transcripts (Fig. 2B), suggesting that the majority of cells producing ca-RNA are unable to produce viral particles. Some wells were positive for one of the three transcripts only (27% [[21], [22], [23], [24], [25], [26], [27], [28], [29], [30], [31], [32], [33]] *gag* ca-RNA only, 0% [0–3] *tat-rev* ca-RNA only, and 4% [[2], [3], [4], [5], [6]] cf-RNA only), which could be explained by different activation status of the infected cells at the time of nucleic acid extraction, distinct expression kinetics of the different transcripts, or deletions or other proviral sequence variation.

**Fig. 3:**
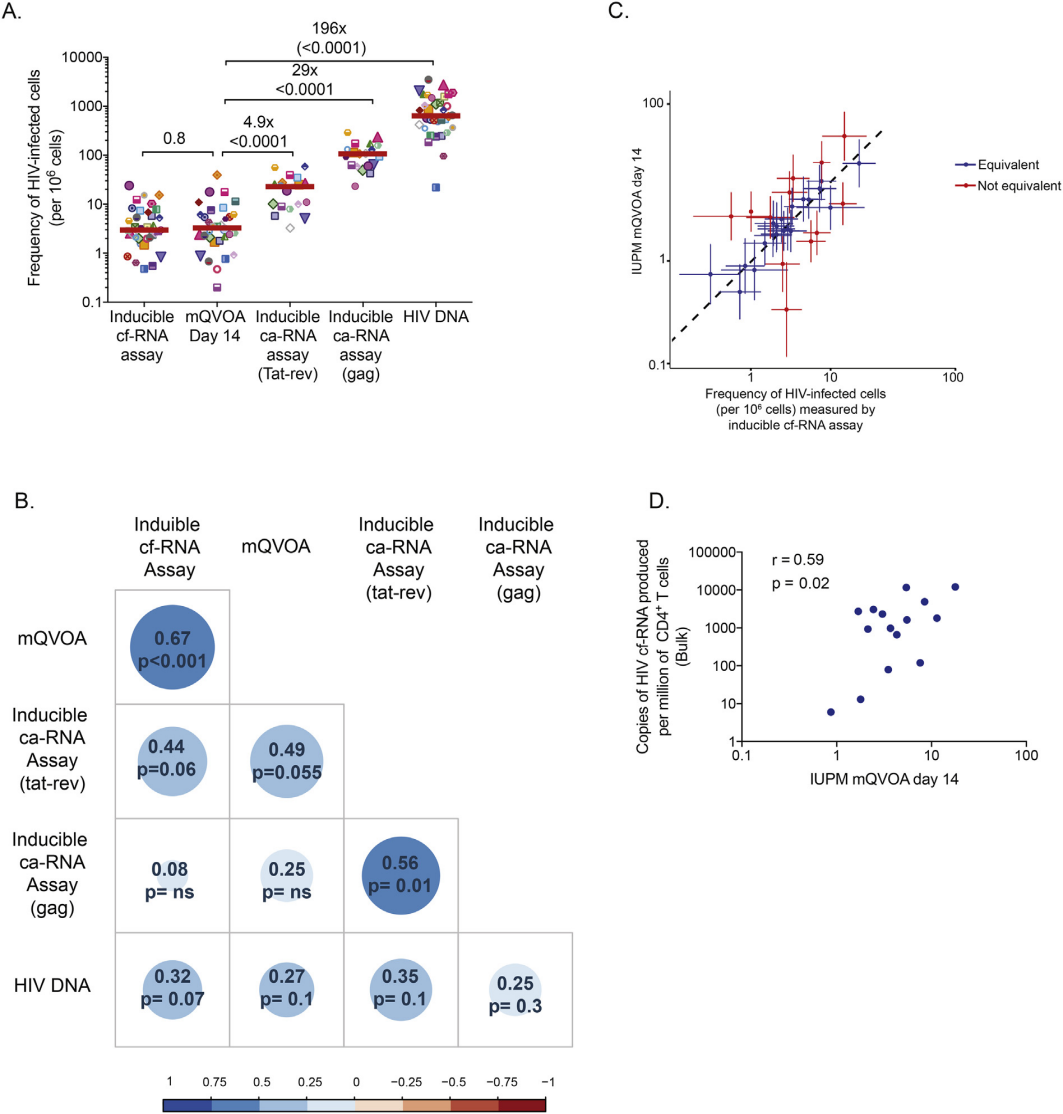
Comparison of HIV reservoir measured by five different assays. (A) The frequency of cells harboring replication competent virus measured by mQVOA was compared with the inducible reservoir, measured as the frequency of cells expressing *gag* ca-RNA, *tat-rev* ca-RNA or production of viral particles upon stimulation. In addition, total HIV DNA (*gag*) was quantified in all samples. Log_10_ transformed individual values and medians are shown. *P*-values were obtained with the student *t*-test for paired data. Fold-changes between mQVOA and other assays are indicated. (B) Correlation matrices between the frequencies of cells harboring HIV reservoir from Panel A are plotted (log_10_ transformed data). Coefficients of correlation and p-values were obtained with Pearson test. The colour and size of the circle show the strength of the correlation between two variables (a bigger circles and darker colors indicate stronger correlations). (C) Correlations between the IUPM (measured by mQVOA at day 14) and the frequency of cells harboring virus with the capacity to produce viral particles (measured by inducible cf-RNA assay). Upper and lower 95% confidence intervals are plotted for both assays. Taking into account the upper and lower 95% confidence intervals, we calculated when mQVOA and inducible cf-RNA assay had equivalent (in blue) or non-equivalent (in red) frequencies. (D) Five million CD4^+^ T cells were stimulated with anti-CD3/CD28 plate bound in bulk for 3 days in the presence of raltegravir. Supernatant was collected and cf-RNA was extracted, quantified by ddPCR and normalized to the number of CD4^+^ T cells. Log_10_ transformed cf-RNA copies per million of CD4+ T cells were correlated with the IUPM measured by mQVOA (day 14). Coefficients of correlation and p-values were obtained from Pearson test.

Despite slight differences in the expression of HIV transcripts, there was a significant correlation between the frequency of cells expressing *gag* ca-RNA and *tat-rev* ca-RNA (*r* = 0.56, *p* = .01, Fig. 3B). Moreover, we observed that the frequency of cells expressing *tat-rev* ca-RNA tended to correlate with the frequency of cells producing viral particles (*r* = 0.44, *p* = .06, Fig. 3B). No correlations were found between the frequency of cells producing *gag* ca-RNA and cf-RNA.

As the inducible RNA assays were run with ddPCR, we were able to calculate the total number of copies of each RNA transcript produced by each cell (Fig. 2C). Despite the fact that the frequency of cells expressing cf-RNA was lower than the frequency of cells producing ca-RNA *gag* or *tat-rev* (Fig. 2A), the total number of copies of cf-RNA produced by each CD4^+^ T cell (558 [290–954] cf-RNA copies/cell) was significantly higher than the total copies of ca-RNA *gag* and *tat-rev* (60 [37–128] *gag* ca-RNA copies/cell and 80 [40–140] *tat-rev ca-*RNA copies/cell, Fig. 2C). No differences were observed in the total number of copies of *gag* or *tat-rev* ca-RNA produced by each CD4^+^ T cell.

As with the mQVOA, we calculated the impact of replicate number on assay precision and accuracy for the inducible RNA assays. We calculated the frequency of infected cells per million using 12, 9, 6, 3, 2 or 1 assay replicates in a subset of individuals, corresponding to 9.00, 6.75, 4.50, 2.25, 1.50 or 0.75 × 10^6^ CD4^+^ T cells assayed, respectively (Supplementary fig. 2). We found no difference in the frequency of cells expressing ca-RNA *gag* and *tat-rev* when calculations were performed with fewer replicates (Supplementary fig. 2A). In contrast, we observed an increase in the frequency of cells producing cf-RNA when using fewer replicates for the calculation (Supplementary fig. 2A). We then calculated the percentage change in the CI95 for each RNA transcript when decreasing the number of replicates (Supplementary fig. 2B). As expected, the CI95 expanded as the number of replicates decreased, which was statistically significant when using fewer than 6 replicates for ca-RNA *gag* and *tat-rev*. In contrast, the CI95 for cf-RNA was significantly wider when using fewer than 9 replicates. Therefore, a minimum of 4.50 and 6.75 × 10^6^ CD4^+^ T cells for ca- and cf-RNA, repectively should be assayed to provide the best balance of required cell numbers with optimal assay precision and accuracy. Of note, the number of cells required for the inducible RNA assays was lower than that used for the mQVOA because of the greater sensitivity of the assays for induced RNA transcripts.

Finally, as with mQVOA, no differences were observed when the inducible RNA assays were run using fresh or frozen samples (Supplementary fig. 2C).

### 3.3 Comparison between mQVOA, inducible RNA assays and HIV DNA

We compared the frequency of HIV infected CD4^+^ T cells measured by mQVOA and the inducible RNA assays (ca-RNA and cf-RNA) with values obtained from HIV DNA quantification by ddPCR. The median frequency of total HIV DNA (*gag*) measured in our cohort was 640 [366–1487] copies of HIV DNA per million CD4^+^ T cells (*n* = 35, Fig. 3A). In contrast, the median frequency of cells harboring replication competent virus measured by mQVOA (day 14) was 3.3 [1.9–6.2] cells/10^6^ CD4^+^ T cells (*n* = 32, Fig. 3A), which was a median of 196 times lower than the frequencies quantified by total HIV DNA (*p* < .0001). The inducible RNA assays showed intermediate frequencies between HIV DNA and mQVOA quantification (Fig. 3A). The frequencies of the inducible reservoir from 19 subjects were 94 [60–132] for *gag* and 16 [[9], [10], [11], [12], [13], [14], [15], [16], [17], [18], [19], [20], [21], [22], [23], [24], [25], [26], [27], [28], [29]] for *tat-rev* ca-RNA cells/10^6^ CD4^+^ T cells, which were 29 and 4.9 times higher, respectively, than the frequencies of replication competent virus measured by mQVOA (p < .0001 and 0.009, respectively). However, they were 6.8 and 40 times lower, respectively, than the frequency of cells harboring total HIV DNA (p < .0001 in both cases). The supernatants collected from the inducible RNA assays permitted the calculation of the frequency of cells releasing viral particles (cf-RNA) after stimulation (*n* = 35), with a median measurement of 2.9 [1.9–6.8] copies/10^6^ CD4^+^ T cells (Fig. 3A). This frequency was 210 times lower than the frequency of cells harboring total HIV DNA (*p* < .0002), but no statistical difference was observed when compared to the frequency of cells carrying replication competent virus (*p* = .5). Moreover, there was a strong statistically significant correlation between mQVOA and the inducible cf-RNA assay (*r* = 0.67, *p* < .001), and mQVOA and the inducible *tat-rev* ca-RNA assay (*r* = 0.49, *p* = .055, Fig. 3B). In contrast, poor correlation was observed between mQVOA and the inducible *gag* ca-RNA assay. None of the assays correlated with total HIV DNA (Fig. 3B).

Since the frequencies of HIV infected cells calculated by mQVOA and inducible cf-RNA assays were in the same range and highly correlated, we performed further analysis to determine if both assays were equivalent. Taking into account the frequency of infected cells with their calculated CI95 in these assays from the same donor, we found that the frequency of infected cells were found equivalent in 20 out of 32 the samples (63%, Fig. 3C). This result suggests that the inducible cf-RNA assay may predict the frequency of cells harboring replication competent virus using a faster and less labor intensive method than the conventional QVOA assay.

Finally, in a subset of individuals (*n* = 15), we evaluated whether the number of viral particles produced by bulk stimulation of 5 × 10^6^ CD4^+^ T cells (Supplementary Fig. 1C) correlated with the frequency of cells harboring replication competent virus. We found a positive correlation between the cf-RNA copies in the supernatant from the bulk stimulation and the mQVOA assay (*r* = 0.59, *p* = .02, Fig. 3D).

## 4 Discussion

The definitive test to evaluate an HIV cure strategy requires the interruption of ART. Therefore, it is necessary to improve current assays to be able to detect precisely a diminution in the size of the HIV reservoir after a cure intervention before considering treatment interruption [33]. A large number of assays have been developed to quantify the frequency of HIV infected cells that persist in ART-suppressed individuals [11,19,22,23,34,35]. Here, we describe several assays that could be used to improve the sensitivity and dynamic range of HIV reservoir measurements.

First, we introduced several modifications to the classical QVOA assay. In most QVOA assays, PHA in the presence of ionomycin or irradiated CD8-depleted PBMCs from uninfected donors are used to activate CD4^+^ T cells from HIV-infected subjects. These protocols cause high cell density in each well that is aggravated when feeder cells are added. In contrast, activation with immobilized anti-CD3/CD28 allows for better control of cell concentration throughout the culture, which is an important factor in prolonged culture. Importantly, this kind of activation has given comparable to superior results compared to alternative protocols for activation of PBMCs from uninfected individuals or PMA/ionomycin [24,36]. PBMCs from at least three uninfected blood donors per assay are needed in the standard QVOA to propagate the infection. The pooled PBMCs from uninfected donors results in high variability between experiments due to different levels of expression of CCR5 molecules and other host cell factors [37], as well as limiting assay throughput. By using the dual co-receptor expressing cell line MOLT-4/CCR5 as feeder cells [19], we reduced cost and labor, and we increased sensitivity, robustness and reproducibility of the assay. The MOLT-4/CCR5 cell line expresses high levels of CD4 and supports infection by both X4-tropic and R5-tropic HIV-1, thereby providing greater uniformity for mQVOA. It is important to note that these rapidly growing cells need to be split and fed twice weekly to optimize culture conditions. In addition, we determined viral outgrowth in MOLT-4/CCR5 by detecting cf-RNA in the culture supernatant by RT-PCR, which allowed us to reduce the number of cell culture days (due to its sensitivity and large dynamic range). It has been shown that ultrasensitive p24 digital ELISA from supernatant of QVOAs may overestimate the size of the replication competent reservoir by detecting biologically irrelevant levels (0.01 pg/ml) of viral protein [38]. Detection of viral cf-RNA in the culture supernatant by RT-PCR is also more sensitive than traditional p24 ELISA. Despite the importance of longitudinal sampling to distinguish viral amplification from low-level viral particles produced by defective proviruses, there are several situations where replication competent virus could be underestimated if only wells that are positive at all time points are counted. First, the kinetics of viral amplification can be slower, and therefore the detection of a positive signal might be delayed to the later time points (i.e. day 14). Also, virus may replicate poorly or fail to sustain propagation in the conditions in vitro (i.e. only detectable at day 7) but be able to propagate readily in vivo. Therefore, the cumulative IUPM would be a better approximate of the replication competent reservoir. However, we cannot exclude that the detection of cf-RNA at day 7 and not day 14 may reflect production of viral particles that cannot propagate.

The frequency of cells harboring replication competent viruses varied across individuals ranging from 0.2 to 39.6 cumulative IUPM (median frequency of 5.8 [2.8–11.4] IUPM), which is higher than values reported for standard QVOA [11,39] or other modified QVOAs [20] (Supplementary Table 1). Despite the improvement in the mQVOA, we cannot disregard that these assays have been run over the past 15 years, using different cohorts, which may have also affected the IUPM measurements. Using a more stringent IUPM calculation, such as considering only wells that are cf-RNA positive at both time points, the number of cells yielding replication competent virus ranged from 0.2 to 25.8 IUPM (median frequency of 2.1 IQR [0.8–4.6] IUPM), which is still greater than values reported previously [11,39]. Altogether, our results suggest improved sensitivity in assay detection of the replication competent reservoir. Despite this improvement, the sequencing of all intact viral genomes indicates that the ‘real’ size of the reservoir could be closer to 60 per million of 10^6^ CD4^+^ T cells [13,40]. We do not know whether some of these “intact” proviruses are in fact defective, are integrated in chromosomal sites not amenable to induction, or do not replicate well in in vitro assays [41].

In parallel, we aimed to develop similar assays in a limiting dilution format for the concomitant detection of inducible ca-RNA (usRNA and msRNA) and virion production. The main advantage of inducible RNA assays are that there is no need for outgrowth of virus with feeder cells before measurement. These assays are therefore much faster than QVOA (only 3 days) and fewer cells are required. We measured HIV transcription (*gag* usRNA and *tat-rev* msRNA) and virion production (cf-RNA) from the same well, permitting an extensive comparison of RNA species. The differences observed in the quantification of the distinct RNA species may provide important information regarding the transcriptional status of infected cells as well the proportion of transcriptionally competent genomes. The frequency of cells capable of producing *gag* usRNA was higher than that producing *tat-rev* msRNA, similar to other studies [42]. However, *gag* transcripts detected by PCR methods may contain internal deletions or other mutations [13]. Therefore, the frequency of cells producing ca-RNA *gag* might be overestimating the size of the replication competent reservoir. Many defective HIV genomes also have deletions that encompass the *tat* and *rev* genes [[12], [13], [14]]. Therefore the quantification of *tat-rev* RNA reduces the likelihood of measuring proviruses with large internal deletions, suggesting that multiply spliced transcripts may be better surrogate markers for productive infection over unspliced transcripts. Accordingly, we found that *tat-rev* ca-RNA expression, but not *gag* ca-RNA expression, correlated with inducible virion production. However, *tat-rev* transcripts are required, but not sufficient, for the production of viral particles, especially with proviruses with frequent deletions in other areas of the viral genome [12,13]. It was also previously shown that the frequency of cells expressing msRNA was highly correlated with the integrated HIV DNA using the TILDA assay [22], which was not confirmed in this study. Importantly, TILDA assay and the inducible tat-rev caRNA assay showed similar frequencies of cells expressing msRNA after stimulation. Despite the advantages of TILDA assay (no cell extraction required and low cell input), the use of more cells in the inducible RNA assay allows a lower limit of detection and a narrower CI95, which can be useful for studies including elite controllers or individuals treated early after infection.

We found that both ca-RNA species were expressed more frequently than cf-RNA. This could indicate a population of infected cells with defective integrated provirus capable of viral transcription but not subsequent virion assembly [[12], [13], [14]]. Latent infection of CD4^+^ T cells has been attributed to blocks to elongation, distal transcription, and multiple splicing [42]. Our results reinforce that there are additional blocks, which could include nuclear export, protein translation, particle formation and/or release [43]. Alternatively, it is possible that three days of culture may not provide sufficient time for all activated cells with integrated HIV to produce intact virions due to different kinetics of activation. We chose 3 days of culture as this was found to represent peak *gag* ca-RNA production for most of the individuals after plate bound anti-CD3/CD28 stimulation (data not shown). However, we clearly showed that there is already an accumulation of viral particles in the supernatant after three days of culture, despite the lower frequency of cells producing viral particles compared to ca-RNA. Importantly, the number of cf-RNA copies produced by each positive cell was higher than the copies of ca-RNA produced, which may increase the sensitivity of viral particle detection. The longer incubation times (i.e. 7 days) adopted in other similar inducible assays [21] may favor the accumulation of viral particles, but may decrease the frequency of cells expressing ca-RNA because cells might die quickly after the expression of viral RNA. These differences in experimental set up may also explain the lack of correlation between the levels of us-RNA and cf-RNA that was previously described [21].

Importantly, the inducible cf-RNA assay described here showed comparable performance to the mQVOA: both assays are equivalent in >60% of the samples tested. This result suggests that the inducible cf-RNA assay could be a good alternative to mQVOA, with shorter culture set up and lower cell requirements. The release of viral particles upon stimulation is the closest step to a replication competent virus; however, not all virions released in in vitro experiments appear to be replication competent [44]. With the high correlation between the frequency of cells producing cf-RNA and replication competence, our data suggest that a large proportion of virions released are replication competent. Pollack et al. were able to detect HIV cf-RNA in the culture supernatant after in vitro transfection of 5 of the 9 defective HIV proviruses tested; the other defective proviruses were unable to form viral particles [44]. However, the virus production by defective proviruses in their in vitro system was 3 to 4 log_10_ lower than the production of virus from a replication competent provirus. Consequently, most defective proviruses are unable to give rise to significant levels of virion production and cells that do produce virions are thus likely to carry replication competent virus.

The frequency of infected cells in ART-suppressed individuals is much lower than that in in vitro cultures. Therefore, even if the vast majority of proviruses are defective in ART-treated subjects, we might not able to detect the suboptimal virion production from these defective proviruses. Interestingly, the copies of cf-RNA produced upon bulk stimulation also correlate with the IUPM. This result might be important for high throughput assays to test different strategies of latency reversing agents, since the cfRNA assay is faster and less expensive. However, the bulk assay does not permit precise calculation of the frequency of cells being reactivated. Although the inducible cf-RNA assay constitutes an attractive alternative to other methods to quantify the size of the latent HIV reservoir, there are potential limitations associated with the use of this assay. First, we cannot conclude that all viral particles released are replication competent. In addition, it is likely that not all HIV proviruses are reactivated during a single round of stimulation [41], similar to other culture-based assays.

In conclusion, we describe novel inducible RNA assays to measure the size of the HIV reservoir that are quantitative, sensitive and allow for the concomitant measurement of different HIV RNA transcripts and viral production with the great advantage that results can be generated in three days. While inducible cf-RNA may be a correlate marker of replication competence, inducible ca-RNA assays may provide more sensitivity and a greater dynamic range. They also may detect cells that can produce viral antigens even if the virus is not replication competent. This assay may thus be valuable for candidate intervention strategies for two reasons. First, it may correlate with the reduction in assays for replication competent virus when an effective intervention that reduces the reservoir is identified. The inducible ca-RNA assay would thus provide the ability to measure 1–2 log_10_ greater reduction than a QVOA. Second, the expression of HIV antigens even without replication competent virus could be contributing to the generalized immune activation and the concomitant pathologic complications observed even in individuals on suppressive ART. As such, the inducible RNA assay may represent an alternative to existing assays used to evaluate the efficacy of therapeutic strategies aimed at reducing the size of the HIV reservoir.

The following is the supplementary data related to this article.

Supplementary Table 1 Summary of some relevant papers performing QVOA. Supplementary Table 1

Supplementary material Image 1
