# Supplementary material for: Improved assays to measure and characterize the inducible HIV reservoir
Source: eBioMedicine. 2018 Oct 11;36:113–21. doi: 10.1016/j.ebiom.2018.09.036 (PMC6197429; doi:10.1016/j.ebiom.2018.09.036)
Supplement: Supplementary Table 1 — Summary of some relevant papers performing QVOA. [file mmc1.pptx]

## Slide 1
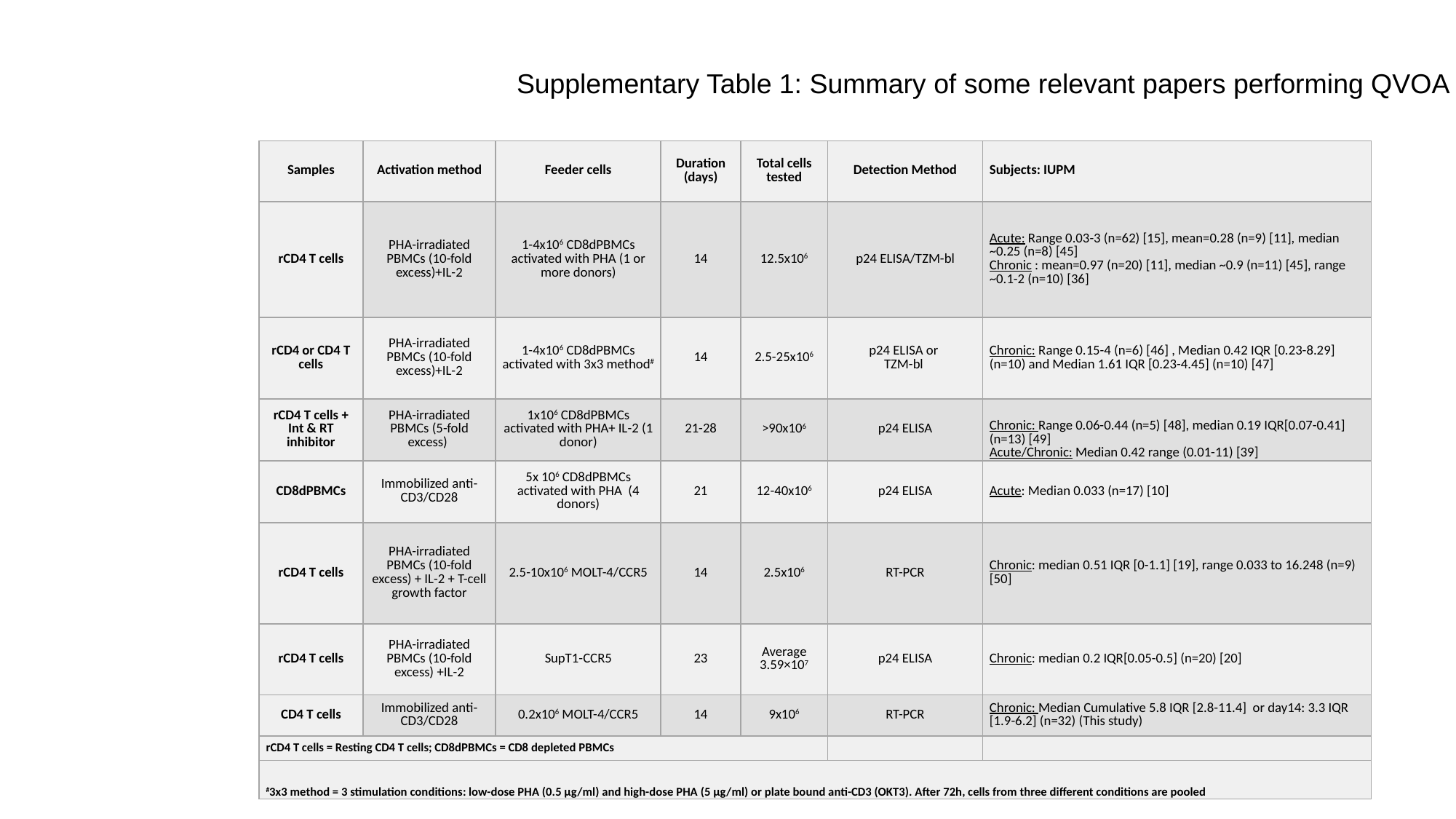

Supplementary Table 1: Summary of some relevant papers performing QVOA
| Samples | Activation method | Feeder cells | Duration (days) | Total cells tested | Detection Method | Subjects: IUPM |
| --- | --- | --- | --- | --- | --- | --- |
| rCD4 T cells | PHA-irradiated PBMCs (10-fold excess)+IL-2 | 1-4x10^6^ CD8dPBMCs activated with PHA (1 or more donors) | 14 | 12.5x106 | p24 ELISA/TZM-bl | Acute: Range 0.03-3 (n=62) [15], mean=0.28 (n=9) [11], median ~0.25 (n=8) [45] Chronic : mean=0.97 (n=20) [11], median ~0.9 (n=11) [45], range ~0.1-2 (n=10) [36] |
| rCD4 or CD4 T cells | PHA-irradiated PBMCs (10-fold excess)+IL-2 | 1-4x10^6^ CD8dPBMCs activated with 3x3 method# | 14 | 2.5-25x106 | p24 ELISA or TZM-bl | Chronic: Range 0.15-4 (n=6) [46] , Median 0.42 IQR [0.23-8.29] (n=10) and Median 1.61 IQR [0.23-4.45] (n=10) [47] |
| rCD4 T cells + Int & RT inhibitor | PHA-irradiated PBMCs (5-fold excess) | 1x10^6^ CD8dPBMCs activated with PHA+ IL-2 (1 donor) | 21-28 | >90x106 | p24 ELISA | Chronic: Range 0.06-0.44 (n=5) [48], median 0.19 IQR[0.07-0.41] (n=13) [49] Acute/Chronic: Median 0.42 range (0.01-11) [39] |
| CD8dPBMCs | Immobilized anti-CD3/CD28 | 5x 10^6^ CD8dPBMCs activated with PHA (4 donors) | 21 | 12-40x106 | p24 ELISA | Acute: Median 0.033 (n=17) [10] |
| rCD4 T cells | PHA-irradiated PBMCs (10-fold excess) + IL-2 + T-cell growth factor | 2.5-10x106 MOLT-4/CCR5 | 14 | 2.5x10^6^ | RT-PCR | Chronic: median 0.51 IQR [0-1.1] [19], range 0.033 to 16.248 (n=9) [50] |
| rCD4 T cells | PHA-irradiated PBMCs (10-fold excess) +IL-2 | SupT1-CCR5 | 23 | Average 3.59×10^7^ | p24 ELISA | Chronic: median 0.2 IQR[0.05-0.5] (n=20) [20] |
| CD4 T cells | Immobilized anti-CD3/CD28 | 0.2x10^6^ MOLT-4/CCR5 | 14 | 9x10^6^ | RT-PCR | Chronic: Median Cumulative 5.8 IQR [2.8-11.4] or day14: 3.3 IQR [1.9-6.2] (n=32) (This study) |
| rCD4 T cells = Resting CD4 T cells; CD8dPBMCs = CD8 depleted PBMCs | | | | | | |
| #3x3 method = 3 stimulation conditions: low-dose PHA (0.5 µg/ml) and high-dose PHA (5 µg/ml) or plate bound anti-CD3 (OKT3). After 72h, cells from three different conditions are pooled | | | | | | |

## Slide 2
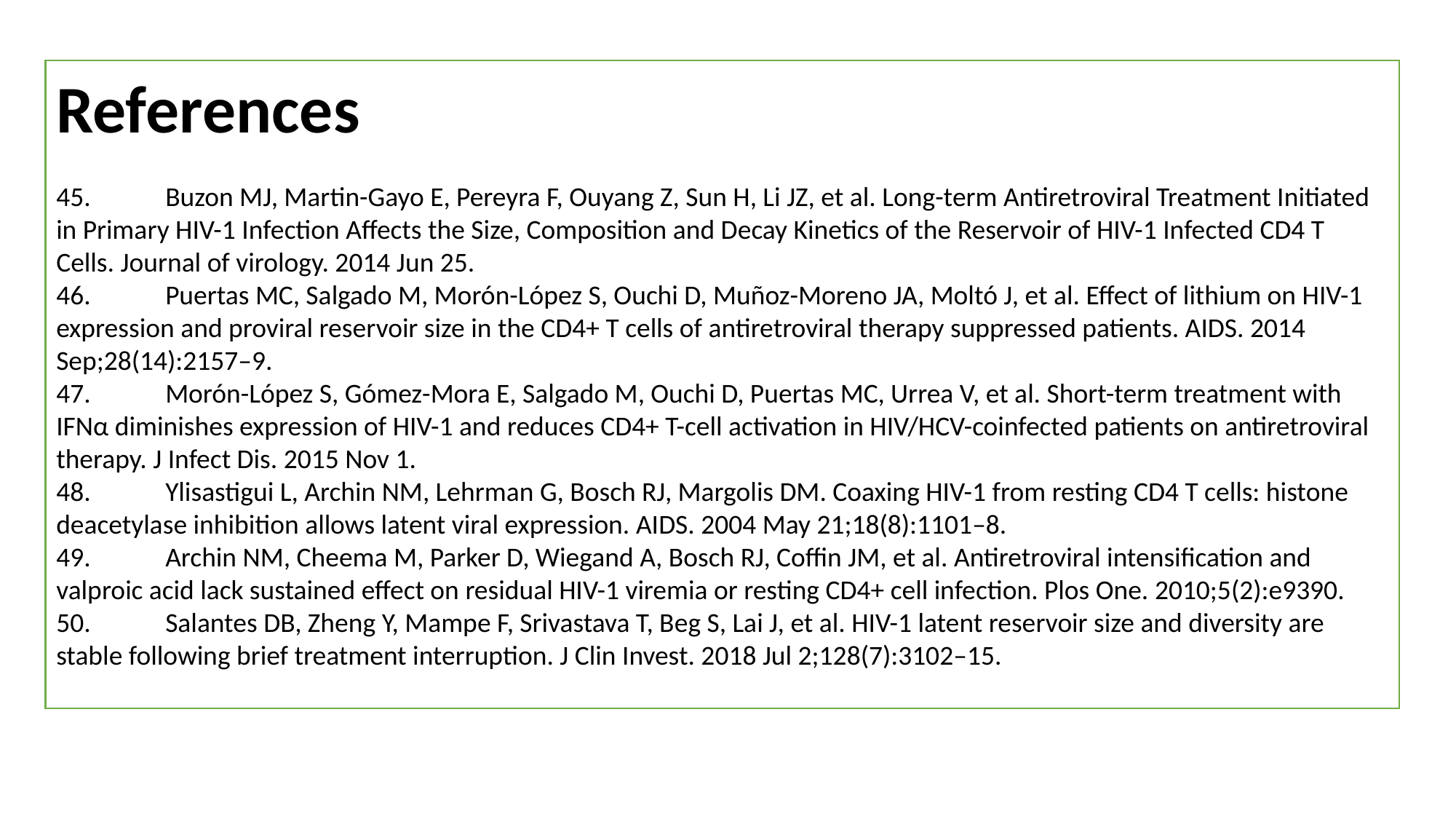

References
45.	Buzon MJ, Martin-Gayo E, Pereyra F, Ouyang Z, Sun H, Li JZ, et al. Long-term Antiretroviral Treatment Initiated in Primary HIV-1 Infection Affects the Size, Composition and Decay Kinetics of the Reservoir of HIV-1 Infected CD4 T Cells. Journal of virology. 2014 Jun 25.
46.	Puertas MC, Salgado M, Morón-López S, Ouchi D, Muñoz-Moreno JA, Moltó J, et al. Effect of lithium on HIV-1 expression and proviral reservoir size in the CD4+ T cells of antiretroviral therapy suppressed patients. AIDS. 2014 Sep;28(14):2157–9.
47.	Morón-López S, Gómez-Mora E, Salgado M, Ouchi D, Puertas MC, Urrea V, et al. Short-term treatment with IFNα diminishes expression of HIV-1 and reduces CD4+ T-cell activation in HIV/HCV-coinfected patients on antiretroviral therapy. J Infect Dis. 2015 Nov 1.
48.	Ylisastigui L, Archin NM, Lehrman G, Bosch RJ, Margolis DM. Coaxing HIV-1 from resting CD4 T cells: histone deacetylase inhibition allows latent viral expression. AIDS. 2004 May 21;18(8):1101–8.
49.	Archin NM, Cheema M, Parker D, Wiegand A, Bosch RJ, Coffin JM, et al. Antiretroviral intensification and valproic acid lack sustained effect on residual HIV-1 viremia or resting CD4+ cell infection. Plos One. 2010;5(2):e9390.
50.	Salantes DB, Zheng Y, Mampe F, Srivastava T, Beg S, Lai J, et al. HIV-1 latent reservoir size and diversity are stable following brief treatment interruption. J Clin Invest. 2018 Jul 2;128(7):3102–15.
