# Supplementary material for: Improved assays to measure and characterize the inducible HIV reservoir
Source: eBioMedicine. 2018 Oct 11;36:113–21. doi: 10.1016/j.ebiom.2018.09.036 (PMC6197429; doi:10.1016/j.ebiom.2018.09.036)

Supplementary figure 1.

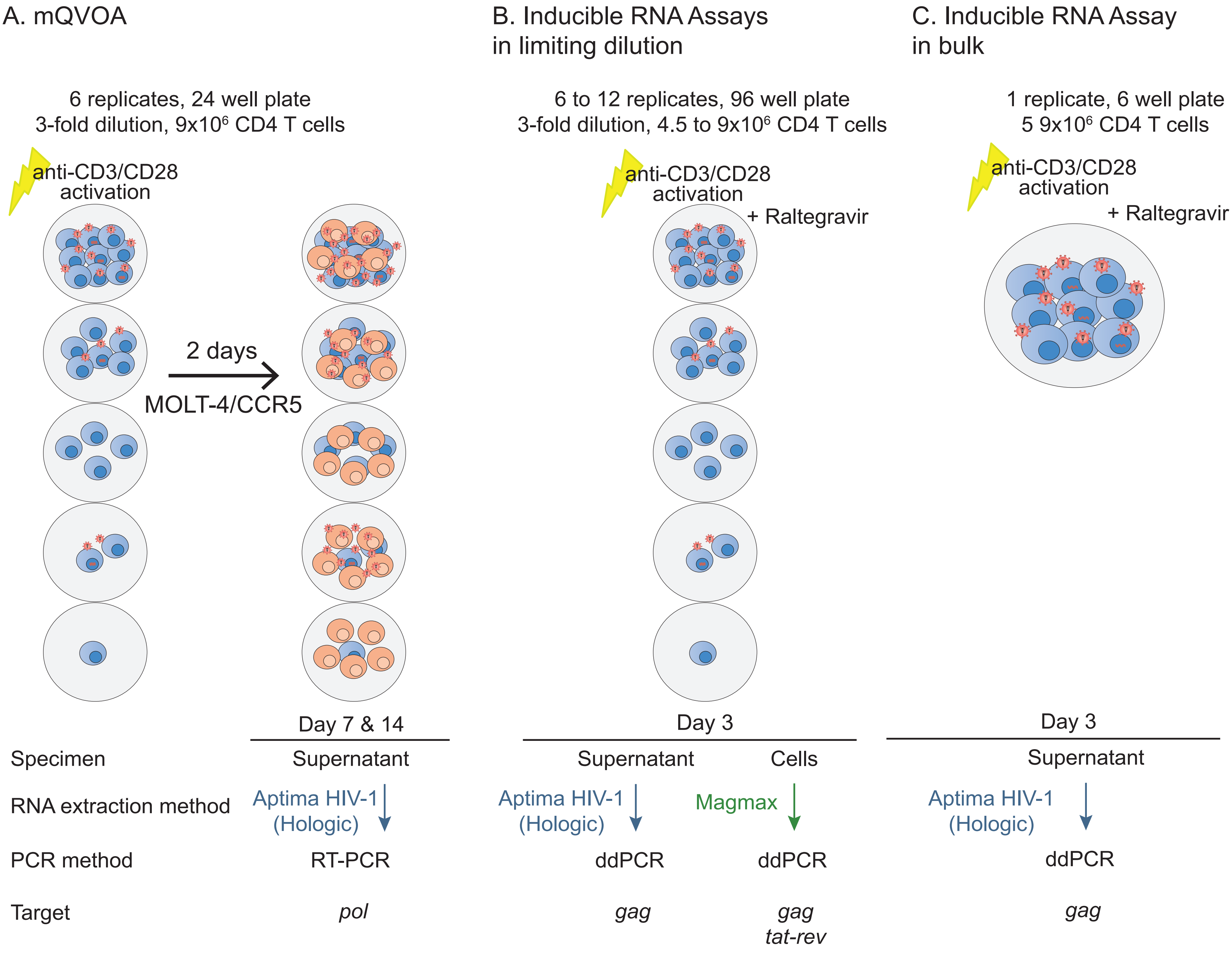

Supplementary Figure 2

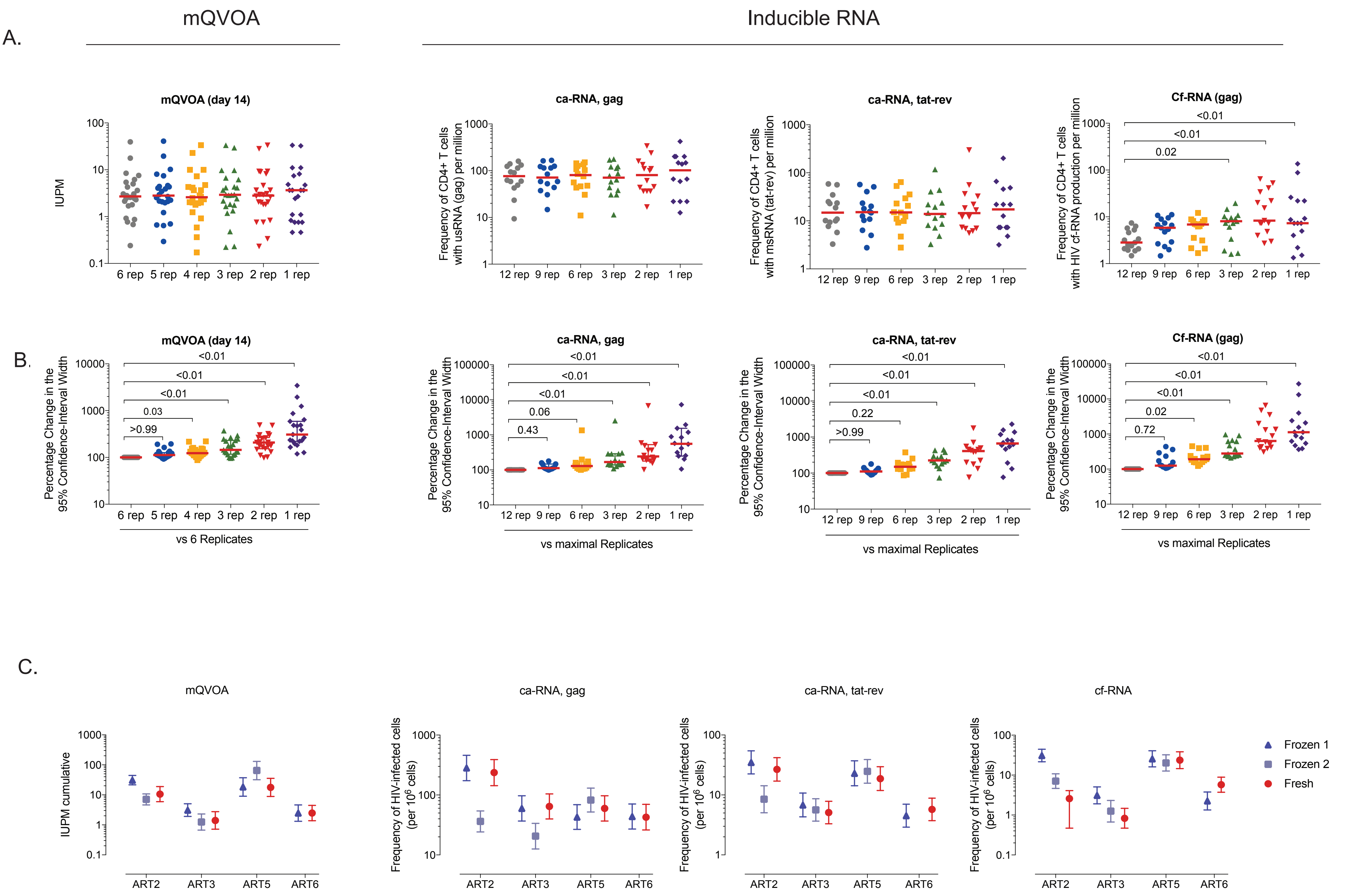

Supplementary figure 3

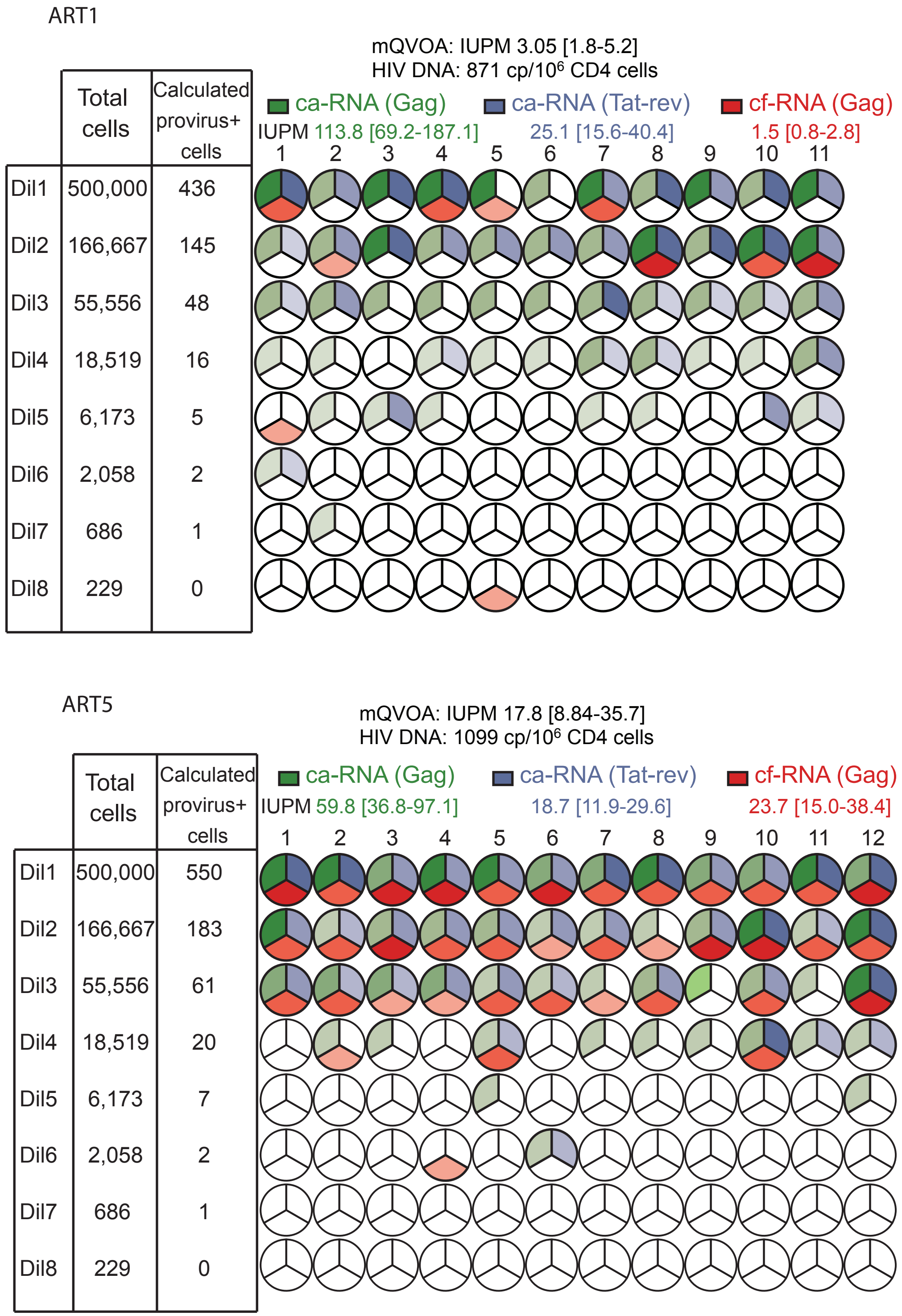

Supplement: Supplementary file 2 — Supplementary material [file mmc2.pdf]
